# Widespread pain and pain intensity in patients with early rheumatoid arthritis. A cross‐sectional comparison between smokers and non‐smokers

**DOI:** 10.1002/nop2.284

**Published:** 2019-04-21

**Authors:** Marie‐Louise Karlsson, Ann‐Charlotte Elkan, Ingiäld Hafström

**Affiliations:** ^1^ Rheumatology Unit Karolinska University Hospital Huddinge Stockholm Sweden; ^2^ Unit of Gastroenterology and Rheumatology, Department of Medicine, Huddinge Karolinska Institutet and Karolinska University Hospital Stockholm Sweden

**Keywords:** nurses, nursing, rheumatoid arthritis, smoking

## Abstract

**Aim:**

The aim was to investigate if smoking status at time for diagnoses of rheumatoid arthritis was associated with pain intensity or pain spread.

**Design:**

A cross‐sectional study conducted in 2012–2013.

**Methods:**

Seventy‐eight patients, of whom 16 were current smokers and 62 never or previous smokers, with newly diagnosed rheumatoid arthritis were assessed as to pain intensity, widespread pain and disease activity.

**Results:**

Of the participants, 56% had unacceptable pain, 77% had spread pain and 28% had chronic widespread pain. There were no differences in pain intensity, widespread pain or chronic widespread pain between smoking status groups. However, there was a positive association between pain intensity and disease activity, *r* = 0.52.

**Conclusion:**

In this study, patients with early rheumatoid arthritis had a high‐frequency unacceptable pain and wide spread pain, irrespective of smoking status. However, we cannot exclude that the inflammatory‐associated pain overshadowed a possible negative effect of smoking.

## 1 INTRODUCTION

In the general population, smoking is associated with widespread pain and fibromyalgia. In patients with rheumatoid arthritis (RA), pain is common, but the association with smoking is uncertain. However, in patients with RA smoking can affect the response to drug therapy. It is therefore important to ascertain if there is a relationship between smoking and pain as soon as possible in the RA disease.

## 2 BACKGROUND

Rheumatoid arthritis is a chronic inflammatory disease with a prevalence of 0.5%–1%. It is characterized of inflammation in small joints in hands and feet, and the main symptoms are pain and morning stiffness from involved joints affecting function and activity. Uncontrolled disease causes joint damage (Scott, Wolfe, & Huizinga, 2010). General symptoms such as fever, cachexia, fatigue and weight loss are also common (Grassi, De Angelis, Lamanna, & Cervini, 1998). Treatment of RA has dramatically improved the last decades with new disease‐modifying antirheumatic drugs (DMARDs) and access to biological agents (Klareskog, Catrina, & Paget, 2009). Despite improved therapy, many patients still have pain. In a Swedish study, 34% of the patients with established RA had widespread pain since more than 3 months, a pain more probably present in smokers than in never smokers (Andersson, Svensson, & Bergman, 2013). Also in other rheumatic diseases with joint involvement, smokers had more widespread pain than non‐smokers (Bremander, Jacobsson, Bergman, Haglund, & Petersson, 2012; Bremander, Petersson, Haglund, Bergman, & Jacobsson, 2012). Furthermore, in populations without any inflammatory rheumatic disease epidemiological studies have shown that smoking is associated with chronic widespread pain and fibromyalgia (Andersson, Ejlertsson, & Leden, 1998; John et al., 2006).

In the last decade, smoking has also been established as a risk factor for developing RA (Klareskog, Padyukov, & Alfredsson, 2007). Furthermore, patients with RA who smoke have a poor response to treatment with tumour necrosis factor blockade (anti‐TNF; Mattey, Brownfield, & Dawes, 2009; Saevarsdottir et al., 2011) and methotrexate (Saevarsdottir et al., 2011). However, as to pain intensity response to anti‐TNF therapy in smokers is inconsistent (Mattey et al., 2009; Soderlin, Petersson, & Geborek, 2012).

Most studies concerning the association between smoking and pain in RA are performed on patients with established disease. There are only few reports addressing the impact of smoking on pain intensity in early RA, and these show divergent results. Thus, two studies report that non‐smokers have less pain intensity than previous smokers and current smokers (Manfredsdottir et al., 2006; Westhoff, Rau, & Zink, 2008), whereas two studies show no difference in pain intensity at disease onset as to smoking status (Ruiz‐Esquide et al., 2011; Soderlin, Petersson, Bergman, & Svensson, 2011). Furthermore, there are no studies exploring if smoking status is associated with differences in pain spread in early RA.

Research question: Is smoking status at time of diagnosis of RA associated with pain intensity and spread of pain and secondly is smoking status or pain associated with disease activity?

## 3 DESIGN

Cross‐sectional study.

## 4 PATIENTS AND METHODS

In all 78 consecutive patients who received a diagnosis of RA for the first time and fulfilled the American College of Rheumatology (ACR) classification criteria (Arnett et al., 1988) were included. All were of Caucasian ancestry and recruited from the day care unit at a rheumatology clinic from January 2012–February 2013. To this unit, most patients in the surrounding area are referred if a RA diagnosis is suggested. At time for diagnosis, the patients should be included into the National Rheumatology Quality Register, which is a web‐based national system that is used to follow incident and prevalent RA cases longitudinally as a part of standard care. Information about patient characteristics including smoking habits, symptom duration, disease activity, disability, pain and treatment are registered.

During the study period, 102 were diagnosed as RA and thus 24 did not participate in the present study as they were not included into the quality register, denied participation or were not asked. These 24 patients did not differ from the study patients as to age, smoking habits or rheumatoid factor (RF) positivity. The 78 patients gave informed consent to participate in the study. As the data are obtained from the quality register, no formal application to ethic committee is required to use the clinical data.

### 4.1 Smoking habits

The patients were registered as current smokers, previous smokers (those who had smoked sometime during the last 30 years) or never smokers. Previous and never smokers were categorized as non‐smokers.

### 4.2 Pain intensity

Pain intensity was measured by a visual analogue scale (VAS). The scale consists of a 100 mm long horizontal line symbolizing degree of pain connected with the question “how much pain did you have today,” anchored with the text “no pain” and “the worst possible pain.” The patient was asked to make a line perpendicular to the VAS line at the point that represented the pain intensity. The score was determined by measuring the distance (mm) between the “no pain” anchor and the patient's mark (Clark, Lavielle, & Martinez, 2003; Price, McGrath, Rafii, & Buckingham, 1983). Unacceptable pain was defined as >40 mm on the pain VAS (Tubach et al., 2012).

### 4.3 Spread of pain

To measure spread pain, a manikin was used with predefined body regions together with description of the 18 regions. Pain was reported by checking the boxes where the patient felt pain. Patients who experienced pain both in the left and right side of the body as well as both above and below the waist were considered to have widespread pain. Further, the patient gave information about the duration of pain. Those with widespread pain more than 3 months were regarded to have chronic widespread pain (CWP; Bergman et al., 2001).

### 4.4 Disease activity and assessments

The disease activity was measured by the composite index Disease Activity Score (DAS) calculated on 28 joints (DAS28). This score includes number of swollen joints, number of tender joints, the patient's global assessment of health measured on visual analogue scale (VAS, 0–100 mm) and erythrocyte sedimentation rate (ESR). The DAS28 score ranges from 0–10.0; <2.6 indicates no disease activity, 2.6–3.2 low disease activity, >3.2–5.1 moderate disease activity and >5.1 high disease activity (Prevoo et al., 1995). ESR was analysed by the Westergren method and RF as well as anti‐cyclic citrullinated (anti‐CCP) antibodies were measured according to the current laboratory standards at the hospital.

### 4.5 Statistics

Non‐parametric statistics were chosen depending on the small sample size. Descriptive data are presented as medians and quartiles (IQR). Differences between groups, current smokers versus never smokers and current smokers versus previous smokers, were assessed using Mann–Whitney *U* test for continues data and Fisher's exact test for dichotomous data. Spearman rho was used for correlations. *p*‐Values < 0.05 were considered statistically significant. Sample size calculation was not performed as prior information was lacking. The program Statistica 12 (Stat soft Scandinavia AB, Uppsala, Sweden) was used for statistical analysis.

## 5 RESULTS

### 5.1 Smoking status

Of the 78 patients, 16 were current smokers, 24 previous smokers and 38 never smokers. The current smokers smoked median 15 cigarettes/day (IQR 8.5–17), and they had smoked for median 43 years (IQR 32–44 years).

### 5.2 Demographics and disease characteristics

Demographics and disease characteristics of the patients grouped per smoking status are shown in Table 1. As can be seen, most patients were women and most of the participants were RF and anti‐CCP positive. Further, the patients had a moderate or high inflammatory activity, measured by DAS28. There were no statistically significant differences in any of these characteristics between the smoking status groups. The symptoms of RA had in 12 patients been present for more than 1 year.

**Table 1:** Demographics and disease characteristics of the patients grouped per smoking status

Characteristics	All patients *N* = 78	Current smokers *N* = 16	Previous smokers *N* = 24	Never smokers *N* = 38
Women, *N* (%)	59 (76)	11 (69)	18 (75)	30 (79)
Age, years	63 (52–72)	59 (50–70)	64 (54–71)	64 (50–73)
Disease duration, months	6 (3–11)	4.5 (3–8)	6 (4–11)	6 (3–11)
RF pos, *N* (%)	59 (76)	15 (94)	17 (71)	27 (71)
Anti‐CCP pos, *N* (%)	63 (81)	14 (86)	19 (79)	30 (79)
DAS28	5.3 (4.7–6.2)	5.1 (4.5–6.2)	5.3 (4.7–6.2)	5.4 (5.0–6.1)
Swollen joints, *N*	8 (5–11)	10 (6–14)	7 (4–10)	8 (5–10)
Tender joints, *N*	7 (4–12)	7 (3–13)	7 (4–12)	8 (4–10)
General health, mm	53 (35–69)	51 (28–71)	50 (42–67)	57 (42–82)
ESR, mm/hr	31 (18–52)	35 (20–66)	37 (18–50)	29 (18–51)

Note

Values are presented as medians (IQR) and numbers (%).

### 5.3 Pain intensity, duration and pain spreading

Pain intensity measured as VAS pain is given in Table 2. There were no statistically significant differences in pain intensity between current smokers and previous or never smokers or between current smokers and non‐smokers. Interestingly, 56% of the patients had a pain intensity more than 40 mm, thus unacceptable pain.

**Table 2:** Pain assessments for all patients and separated in the different smoking groups

	All participants *N* = 78	Current smokers *N* = 16	Previous smokers *N* = 24	Never smokers *N* = 38
Pain intensity	45.5 (17.5–70)	46 (25–74)	50 (17–67)	43 (14–69)
Unacceptable pain	43 (56)	9 (56)	14 (58)	20 (54)
Pain duration	7 (3–22)	4 (2–21)	11 (6–60)	6 (2–20)
Pain for more than 12 months	24 (31)	5 (31)	8 (33)	11 (29)
Spread pain without back	60 (77)	10 (63)	20 (83)	30 (79)
Spread pain including back	32 (41)	4 (25)	10 (42)	18 (47)
CWP	22 (28)	3 (19)	9 (38)	10 (26)

Note

Values are presented as medians (IQR) and numbers (%).

The median pain duration was 7 months but there were large variations in all groups, Table 2. Minimum pain duration was 0.25 months, and maximum was 32 years. A total of 24 participants (31%) had suffered from pain for more than 1 year. There were no statistically significant differences in pain duration or number of patients with pain >1 year between the different smoking status groups.

Nor when women were analysed separately were there any statistically significant differences in pain intensity but as to pain duration it was a difference between smokers and ex‐smokers. Smokers had a pain duration for 4.50 months (IQR 2–18) versus former smokers 11 (IQR 6–60) months, *p* = 0.049.

There was a high prevalence of spread pain, 77% (Table 2), but no statistically significant difference between smoking status groups. The findings were similar when only women were analysed (data not shown).

The frequency of chronic widespread pain, CWP, (pain duration of more than 3 months) was in all patients 28%, in current smokers 19%, in previous smokers 38% and in never smokers 26%, not significantly different.

### 5.4 Pain and disease activity

As smoking status was not associated with pain intensity, we further analysed if pain intensity was associated with disease activity and found such an association with DAS28, *r* = 0.52, *p* = 0.05. There was no statistically significant difference in DAS28 between the participants who had widespread pain and those without this kind of pain.

## 6 DISCUSSION

This study revealed that many patients (56%) had unacceptable pain and a high prevalence had widespread pain (77%) and chronic widespread pain (28%) at the time for diagnose of RA. Neither pain intensity and duration nor pain spread differed between current smokers, previous smokers and never smokers. Further, pain intensity was significantly associated with DAS28, but no association was found between widespread pain and DAS28.

An important observation was that more and half of the participants had pain intensity of more than 40 mm, measured by the VAS scale. The pain intensity in this study was of the same magnitude as in most previous studies in patients with new onset of RA (Ruiz‐Esquide et al., 2011; Soderlin et al., 2011; Westhoff et al., 2008) even if higher pain intensity has been reported (Manfredsdottir et al., 2006).

The intensity of pain at the onset of RA is usually explained by joint inflammation that gives rise to nociceptive pain. A support for such a relationship was also demonstrated here by the correlation between pain intensity and DAS28, which suggests that higher disease activity is associated with more intensive pain. Further, DAS28 did not differ between smoking groups. Several earlier studies report similar disease activity in different smoking groups (Ruiz‐Esquide et al., 2011; Soderlin et al., 2011; Westhoff et al., 2008), even if other describe that smokers had higher disease activity (Manfredsdottir et al., 2006; Papadopoulos et al., 2005).

In the women, the shorter pain duration in current smokers compared with previous smokers can only be speculated on. A possible explanation is that the known analgesic effects of nicotine affect the pain system at various levels by involving the activation of nicotinic acetylcholine receptors (Ditre, Brandon, Zale, & Meagher, 2011). Thus, in studies on non‐rheumatic individuals decreased pain perception has been shown in smokers versus previous smokers (Fertig, Pomerleau, & Sanders, 1986; Girdler et al., 2005) and smoking has been shown to have a distraction effect on pain (Hooten et al., 2011).

To our knowledge, there are no previous studies on the prevalence of widespread pain in newly diagnosed RA. In established disease, though, the frequency of chronic widespread pain was about the same (34%) as in the present study. However, in patients with established disease, smokers had more chronic widespread pain than never smokers, but the areas of chronic wide spread pain also included the back pain and not only the four quadrants (Andersson et al., 2013).

In contrast to reports by Andersson et al. (2013), Bremander, Jacobsson, et al. (2012) and Bremander, Petersson, et al. (2012), we found no difference in spread pain between smoking status groups. A possible explanation could be that the present patients had early RA and not established disease with long duration. It is known that smoking is a predictor for bad treatment response to, that is, DMARDs and TNF‐alpha inhibitors (Abhishek, Butt, Gadsby, Zhang, & Deighton, 2010; Canhao et al., 2012; Saevarsdottir et al., 2011), and high disease activity is associated with worse pain.

### 6.1 Strengths and limitations

The strength of this study is its prospective nature, and that most patients with early RA in the area are referred to the department. These facts together with that all patients performed the pain drawing and all but one the pain intensity scale (VAS pain) imply the results to be representative for patients with newly diagnosed RA.

The limit of the study is the small number of participants and the absence of information of use of analgesic's, depression, education, marital status and BMI. Since this study was the first to study widespread pain at newly diagnosis of rheumatoid arthritis, it was not possible to calculate sample size.

## 7 CONCLUSION

In this study of patients with early RA, there was a high frequency of patients with unacceptable pain and wide spread pain, irrespective of smoking status. However, pain intensity correlated positively with disease activity, why we cannot exclude that the inflammatory‐associated pain overshadowed a possible effect of smoking. Additional studies are needed to solve this issue as an important issue in rheumatology care is to help smokers quit smoking.

## CONFLICT OF INTEREST

The authors declare no competing interests.

## AUTHORS CONTRIBUTIONS

M‐LK participated in planning the study, recruited the patients, gathered the data, performed the statistical analyses and drafted the manuscript. A‐CE participated in the statistical analyses and critically revised the manuscript. IH participated in planning the study design and data interpretation and helped to draft the manuscript. All authors approved the final manuscript.
